# Susceptibility of Shy Students to Internet Addiction: A Multiple Mediation Model Involving Chinese Middle-School Students

**DOI:** 10.3389/fpsyg.2019.01275

**Published:** 2019-05-29

**Authors:** Yang Yu, Hong Sun, Fengqiang Gao

**Affiliations:** ^1^Department of Psychology, Shandong Normal University, Jinan, China; ^2^Jinan Technician College, Jinan, China

**Keywords:** shyness, internet addiction, cognitive flexibility, self-regulation, self-inconsistency

## Abstract

Recent studies found that some personality traits (e.g., impulsivity, sensation seeking) are frequently related to Internet addiction. In line with previous studies, this study aimed to determine whether shy students readily develop Internet addiction and to identify the causes of their developing Internet addiction. Specifically, this study examined the mediating roles of cognitive flexibility, self-regulation, and self-inconsistency in linking shyness and Internet addiction. A total of 1301 middle-school students in Shandong Province, East China, completed the relevant scales. Correlation analysis revealed that shyness was positively correlated with self-inconsistency and Internet addiction and negatively correlated with self-regulation and self-inconsistency. Cognitive flexibility, self-regulation, and self-inconsistency played fully mediating roles in the relationship between shyness and Internet addiction. The results indicate the significance of shyness-sensitivity for Internet addiction and suggest that cognitive and coping abilities as well as social adjustment factors should be considered when designing interventions to help shy students overcome Internet addiction.

## Introduction

Shyness is defined according to characteristics such as not wishing to participate in social activities and blushing when communicating. It involves experiencing reticence and anxiety in social situations. Although shy persons may wish to engage in conversations, their approach motivations are often restrained because of anxiety and hesitation (Coplan et al., 2004). Due to its contradictory approach–avoidance motivation (high approach-avoidance motivation), shyness differs from other social withdrawal phenomena such as social silence (low approach-avoidance motivation), and behavioral inhibition (low approach and high avoidance motivation) (Asendorpf, 1990; Rubin et al., 2009; Coplan and Rubin, 2010).

In traditional Chinese societies, “the golden mean of the Confucian school” is emphasized; consequently, behavioral restraint and wariness are considered positive traits. However, since the 1980s, China has developed into a competitive, market-oriented society due to extensive economic reform. Self-expression, independence, and initiative taking are increasingly highly valued, whereas shyness and reticence have become less accepted (Yang et al., 2015). Thus, in the context of urban Chinese culture, shy individuals have been found to experience adjustment difficulties, including internalizing and externalizing problems (Chang et al., 2005; Yang et al., 2015). For instance, a four-wave longitudinal study investigated the bidirectional relations between shyness and social adjustment in urban Chinese children and found that shyness positively contributed to social, educational, and psychological difficulties over time, with the most consistent effects involving peer preference and loneliness (Yang et al., 2015). Studies have argued that the socialization and academic outcomes of shy individuals are related to emphasis on assertiveness, competitiveness, and self-expression in society (Greenfield et al., 2006). The world has become a “global village” because of economic globalization. Therefore, communication skills are crucial for economic and social survival. Thus, in the contemporary society, shy individuals may increasingly experience social adjustment problems. Among such problems, Internet addiction has recently attracted much scientific attention (Davis, 2001; Scealy et al., 2002; Lavin et al., 2004; Orr et al., 2009; Selfhout et al., 2009; Young and Lo, 2012; Huan et al., 2014).

Nowadays, using the Internet for learning and entertainment has become one of the most popular activities in many countries, including China. As reported, the number of Internet users in China had reached 829 million by December 2018 (China Internet Network Information Center [CNNIC], 2019). With the increase in Internet use, Internet addiction has become a marked problem in young people. This concern has emerged following recent international research on adolescents that identified a heightened negative impact of Internet addiction on their health, social behavior, and academic performance (Kaess et al., 2014; Zhang et al., 2015). In early Internet addiction studies, Young (1996) developed an eight-item diagnostic questionnaire (DQ) and provided criteria for addictive Internet use. Other researchers used pathological Internet use (PIU; Morahan-Martin and Schumacher, 2000) or Internet dependency (Scherer, 1997) as an index of Internet use associated with interpersonal problems, distress, tolerance symptoms, and mood alteration. In the present study, the term “Internet addiction” was used to collectively indicate phenomena related to Internet misuse.

Individuals, especially those who have difficulty with social interactions, can now choose the medium that makes them feel most comfortable communicating with others or that complements their lack of communication skills (Hammick and Lee, 2014). Unsurprisingly, numerous studies have shown that because of greater anonymity and fewer non-verbal cues, shy individuals prefer using the Internet to face-to-face interactions to socialize with others and combat loneliness (Bardi and Brady, 2010; Hammick and Lee, 2014; Huan et al., 2014; Nelson et al., 2016). A few studies have revealed that the Internet may assist shy individuals in building relationships, and providing them with more opportunities to develop social skills (Scharlott and Christ, 1995; Saunders and Chester, 2008). However, more research has indicated that the preference of shy individuals for the Internet as a means of communication and entertainment often leads to Internet addiction because it is difficult for such individuals to effectively deal with its negative effects (Chak and Leung, 2004; Huan et al., 2014; Nelson et al., 2016). This study aimed to determine whether shy students readily develop Internet addiction and to identify the possible causes of their developing Internet addiction in mainland China.

Of the many factors influencing the relation between shyness and Internet addiction, deficient cognitive and coping abilities may be crucial factors affecting the proneness of shy students to Internet addiction. Excessive self-concern or behavioral inhibition may prevent shy students from availing opportunities to develop their cognitive and coping abilities through participation in social activities. However, these cognitive and coping abilities may be necessary to prevent Internet addiction. Consistent with this hypothesis, researchers found cognitive appraisal bias (i.e., heighten estimation of social risk and deflated self-efficacy) or maladaptive cognitions mediated the relationship between shyness and internet addiction, respectively (Davis, 2001; Young and Lo, 2012). In the present study, we specifically focused on the mediating roles of cognitive flexibility and self-regulation in the association between shyness and Internet addiction. Cognitive flexibility refers to the ability to adjust cognitive processing strategies in order to adapt to new environments (Cañas et al., 2003). Higher cognitive flexibility enables a person to distract attention from negative phenomena, actuate conceptual change, use working memory flexibly, and generate a selective strategy. Self-regulation is a multicomponent construct (e.g., physiological regulation, social-emotional regulation, behavioral regulation) involving people’s ability to effectively plan or modulate their behavior in consideration of an adaptive consequence (Montroy et al., 2016). In the context of emotion, self-regulation refers to attempts to alter negative emotional experiences. Difficulties with emotion regulation are believed to be risk factors for Internet addiction (Hormes et al., 2014).

Eysenck proposed that worry or self-preoccupation, which is characterized by concerns regarding evaluation and expectation of aversive consequences, impairs efficient functioning of the goal-directed attentional system and increases the extent to which processing is influenced by the stimulus-driven attentional system. Furthermore, self-preoccupation increases individuals’ attention to threat-related stimuli (Eysenck et al., 2007). This theory can satisfactorily explain behavioral patterns in shy individuals, as shyness involves excessive worry or self-preoccupation regarding evaluation by others (Yu et al., 2019), punishment, or non-reward cues (Xu et al., 2009). The impaired attentional control system of shy individuals causes low cognitive flexibility, which may be a vital cause of Internet addiction. In a social context, particularly where punishment, evaluation, or social cues provided by others are prominent (e.g., school), an impaired goal-directed attentional system may weaken cognitive flexibility in shy individuals, hinder the flexible use of working memory, or prevent the generation of selective strategies. Consequently, effectively planning or modulating the behaviors of such individuals to complete goal-directed work and study is difficult (difficulties with self-regulation). However, the rich and colorful network resources (e.g., online games, chat room), which can easily capture attention automatically, may be highly agreeable to the increased stimulus-driven attentional system of shy individuals. Consequently, such individuals become immersed in a virtual network world. Furthermore, the defective attention system of shy students causes an increase in attention to threat-related stimuli, and this may render such individuals unable to treat novel stimuli or punishment or non-reward cues flexibly, disable them from distracting themselves from negative social cues, or exaggerate negative affectivity. Moreover, exaggerated negative affectivity or negative social cues may cause further failure of emotional regulation, and this may also be crucial to the development of Internet addiction (Hormes et al., 2014). Overall, low cognitive flexibility may cause failure of self-regulation (particularly difficulties with emotional regulation), which can further increase the likelihood of Internet addiction in shy individuals.

Another important factor underlying the relation between shyness and Internet addiction is likely to be social maladjustment. Considering self-defects and deficient cognitive and coping abilities (e.g., cognitive flexibility, self-regulation), researchers have reported that shyness is associated with a series of social–emotional and school adjustment problems (Chang et al., 2005; Yang et al., 2015). Consequently, shy individuals may develop alcohol dependence or become more aggressive as the result of poor social adaptation (Maroldo, 1986; Hutteman et al., 2009).

Similar to alcohol dependence and aggressiveness (Maroldo, 1986; Hutteman et al., 2009), Internet addiction is likely to be the product of social maladjustment of shy individuals. Consistent with this hypothesis, researchers found loneliness or perceived friendship quality mediated the relationship between shyness and Internet addiction, respectively (Selfhout et al., 2009; Huan et al., 2014). According to uses and gratifications theory, audiences differ in the gratifications they seek from media. The gratifications can be classified as seeking diversion (escape from problems, emotional release), developing personal relationships, establishing personal identity, and acquiring information (McQuail et al., 1972; Sheldon, 2008). Additionally, the social compensation hypothesis proposes that the anonymity and reduced cues that characterize the Internet might stimulate online self-disclosure; thus, the Internet may have enhanced benefits for socially withdrawn individuals (McKenna and Bargh, 2000; Sheldon, 2008). These two theories suggest that the tendency of shy students to exhibit Internet addiction is likely to be the product of social failure. That is, when shy students develop internalizing problems (e.g., negative emotions, self-inconsistency) because of social failure, or become aware that their insufficient ability to regulate their emotion prevents them from encountering social reality, they may compensate themselves or choose to release their emotions through the Internet. In the present study, self-inconsistency, which is a crucial concept in Rogers’ personality theory (Rogers, 1959), was considered an indicator of social maladjustment. Self-inconsistency reflects ability and emotion evaluation, self-consistency, and a sense of helplessness. A high degree of self-inconsistency can be considered an outcome of social maladjustment. We suggested that shy students may compensate for high levels of social maladjustment though excessive use of the Internet.

**FIGURE 1:**
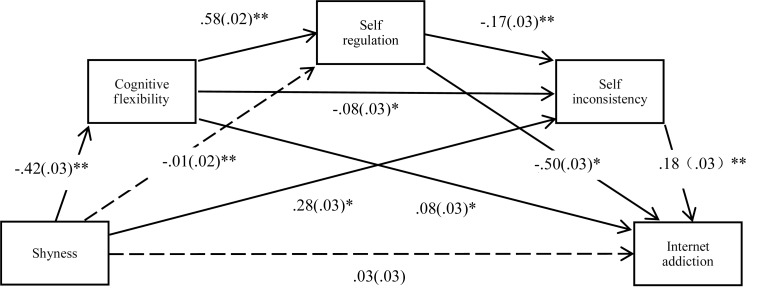
Multiple mediation model for relations among shyness, cognitive flexibility, self-regulation, self-inconsistency, and Internet addiction. path values are the standardized regression coefficients (standard errors), *N* = 1301, ^∗^*p* < 0.05, ^∗∗^*p* < 0.01.

Notably, shy individuals may develop Internet addiction because of their deficient cognitive and coping abilities and social maladjustment. In this study, the relation between shyness and Internet addiction in Chinese middle-school students was investigated, with cognitive flexibility, self-regulation, and self-inconsistency applied as the mediating variables. Among the mediator variables, cognitive flexibility and self-regulation are indicators of cognition and coping ability. Given that poor cognitive flexibility may cause difficulties in self-regulation and social maladjustment, cognitive flexibility may be a relatively superior mediator. Given that poor cognitive flexibility and self-regulation may be vital causes for social maladjustment, self-inconsistency may be an inferior mediator indicating social maladjustment among shy students. Figure 1 depicts the mediation effect model.

## Materials and Methods

### Participants and Procedure

Middle-school students (1320 non-clinical participants; mean age = 12.51 ± 1.00 (*M* ±*SD*); 50.2% female) were recruited from five schools in Shandong Province, East China, using a cluster sampling method. A battery of self-report questionnaires (see “Questionnaires” section) was administered collectively during regular class lasting approximately 30 min. All students provided informed consent and received small gifts for their participation. A total of 1301 valid questionnaires (valid rate: 98.6%) were collected and analyzed. The distribution of the participants is shown in Table 1. The mediating roles of cognitive flexibility, self-regulation, and self-inconsistency in the relationship between shyness and Internet addiction were tested using the PROCESS macro for SPSS (Hayes, 2013). Model 6 was selected. According to the bootstrap method proposed by Preacher and Hayes (2004) and then developed by Hayes (2013), the mediation effect (with *n* = 5,000 bootstrap resamples) is observed when the bias-corrected confidence interval (95%) of the indirect effect does not pass through zero.

**Table 1:** Demographic variables and frequency distribution.

				Missing value	Sum
Gender	Boys	Girls			
	646(49.7%)	653(50.2%)		2(0.2%)	
Grade	Grade 1	Grade 2	Grade 3		
	654(50.3%)	450(34.6%)	197(15.1%)	0	
School location	City	Village			
	539(41.4%)	762(58.6%)		0	
Only child	Yes	No			
	538(41.4%)	736(56.6%)		27(2.1%)	1301


### Questionnaires

#### Revised Cheek and Buss Shyness Scale (RCBS)

Cheek (1983) developed a shyness scale (13 items) to measure individuals’ uncomfortable reaction and behavioral inhibition in front of others on a 5-point Likert-type scale (1 = *strongly disagree*; 5 = *strongly agree*). For instance, “I feel tense when I am with people I do not know well.” The scale has been revised to produce a Chinese version and has been widely used among college and middle school students (Zhao et al., 2012; Li et al., 2013). A higher score represents a higher degree of shyness. In this study, the Cronbach’s α was 0.74.

#### Cognitive Flexibility Inventory-Revised

Martin and Rubin (1995), Martin et al. (1998) developed the Cognitive Flexibility Inventory (12 items, three factors) to measure individuals’ selective cognitive consciousness on a 6-point Likert-type scale (1 = *strongly disagree*; 6 = *strongly agree*). For instance, “I can express an idea in many different ways.” The scale has been revised to produce a Chinese version and has been widely used in Chinese contexts (Qi et al., 2013). A higher score represents a higher degree of cognitive flexibility. In this study, the Cronbach’s α was 0.73.

#### Self-Regulation Questionnaire

The Chinese Self-regulation Questionnaire (38 items, three factors) was adopted to measure ability to take initiative in setting goals, use strategies and resources effectively, and achieve established goals on a 4-point Likert-type scale (1 = *strongly disagree*; 4 = *strongly agree*). For instance, “I usually make a schedule of my daily activities.” The questionnaire has been used in adolescents (Gao and Zhang, 2013). A higher score represents a higher degree of self-regulation. In this study, the Cronbach’s α was 0.91.

#### Self-Inconsistency Scale

The Chinese Self-inconsistency Scale (16 items) was adopted to reflect ability and emotion evaluation, self-consistency, and a sense of helplessness on a 5-point Likert-type scale (1 = strongly disagree; 5 = strongly agree) (Walker et al., 1960; Rogers, 1961). For instance, “In many cases, I have to question my ability.” A high level of self-inconsistency can be considered an outcome of social maladjustment. This scale has been widely used in adolescents (Zhou, 2015). In this study, the Cronbach’s α was 0.74.

#### Internet Addiction Test

The Internet Addiction Test (20 items), was used to measure the level of Internet addiction on a 5-point Likert-type scale (1 = *few*; 5 = *always*) (Young, 1996; Young and de Abreu, 2011). For instance, “How often do you snap, yell, or act annoyed if someone bothers you while you are online?” It has been validated in many languages, including English, Greek, Italian, and Chinese and widely used among college and middle school students (Lu and Yeo, 2015). In this study, the Cronbach’s α was 0.90.

## Analysis and Results

The descriptive statistics of all the variables and Pearson’s *r* correlations between the variables are presented in Table 2. The results indicate that shyness was positively related to self-inconsistency and Internet addiction, whereas negatively related to cognitive flexibility and self-regulation. According to Young’s diagnostic criteria (Young, 1999; Liu, 2008), 458 students (35.20%) did not exhibit Internet addiction disorder (IAD; score ranging 0–30), 579 students (44.50%) exhibited mild Internet addiction disorder (score ranging 31–49), 255 students (19.60%) exhibited moderate Internet addiction disorder (score ranging 50–79), and 9 students (0.69%) exhibited severe Internet addiction disorder (score ranging 80–100).

**Table 2:** Means (*M*), standard deviations (*SD*), and correlations between variables (*N* = 1301).

	*M*	*SD*	1	2	3	4
(1) Shyness	34.48	8.29	1			
(2) Cognitive flexibility	42.21	7.88	–0.42**	1		
(3) Self-regulation	95.68	13.85	–0.25**	0.58**	1	
(4) Self-inconsistency	16.73	4.62	0.36**	–0.29**	–0.28**	1
(5) Internet addiction	38.28	13.66	0.19**	–0.28**	–0.51**	0.31**


*^∗^p < 0.05, ^∗∗^*p* < 0.01; *M* = total score.*

Mediation analyses were then conducted. Figure 1 shows the mediation effect model and path coefficients. Except for two (shyness→Internet addiction, and shyness→self regulation), all path coefficients in the model were significant. Table 3 shows the 95% confidence intervals of all path coefficients. Except for indirect effect paths 5 and 6, the bias-corrected confidence intervals (95%) of the other indirect effect paths did not pass through zero, indicating that the mediating effects were significant. After the mediating variables had been controlled, shyness did not show a significant direct predictive effect on Internet addiction, indicating that the mediating variables played fully mediating roles in the relationship between shyness and Internet addiction.

**Table 3:** Bootstrap analysis results for mediating effects.

Indirect impact path	Average indirect effect	95% confidence interval	
		lower	upper
(1) shyness→cognitive flexibility→Internet addiction	–0.03	–0.06	–0.005
(2) shyness→cognitive flexibility→self-regulation →Internet addiction	0.12	0.10	0.14
(3) shyness→cognitive flexibility→self-inconsistency →Internet addiction	0.01	0.001	0.01
(4) shyness→cognitive flexibility→self-regulation→self-inconsistency →Internet addiction	0.01	0.004	0.01
(5) shyness→self-regulation→Internet addiction	0.004	–0.02	0.03
(6) shyness→self-regulation→self-inconsistency→Internet addiction	0.0002	–0.001	0.002
(7) shyness→self-inconsistency→Internet addiction	0.05	0.03	0.07


## Discussion

To explain the mechanisms underlying the relation between shyness and Internet addiction, the present study proposed and tested a theoretical model based on a survey involving 1301 Chinese middle-school students in East China. The results revealed that shyness was positively correlated with self-inconsistency and Internet addiction, whereas negatively correlated with cognitive flexibility and self-regulation. Moreover, cognitive flexibility, self-regulation, and self-inconsistency played fully mediating roles in the relationship between shyness and Internet addiction. The results indicated that shy students are prone to Internet addiction and suggest that cognitive and coping abilities as well as social adjustment factors should be considered when designing interventions to help shy students overcome Internet addiction.

### Role of Cognitive Flexibility

Cognitive flexibility refers to the ability to adjust cognitive processing strategies to adapt to new environments (Cañas et al., 2003). As expected, cognitive flexibility plays an important role in the relationship between shyness and Internet addiction. The results indicate that cognitive flexibility influences the relationship between shyness and Internet addiction through two modes. The positive predictive role of cognitive flexibility in the relationship between shyness and Internet addiction operates through self-regulation and self-inconsistency (indirect positive effects, see Figure 1 and Table 3); however, after controlling for self-regulation and self-inconsistency, it was revealed that cognitive flexibility plays a negative predictive role in the relationship between shyness and Internet addiction (indirect negative effects, see Figure 1 and Table 3). The two modes are discussed separately as follows.

First, low cognitive flexibility in shy students can positively predict Internet addiction through self-regulation and self-inconsistency. As described in the Introduction section, Eysenck proposed that excessive worry regarding evaluation and expectation of aversive consequences impairs the attentional control system, and thus reduces the effect of the goal-directed attentional system and increases the effect of the stimulus-driven attentional system. The impaired attentional control system of shy individuals causes low cognitive flexibility, which makes them unable to flexibly plan or modulate their behaviors to fulfill goal-directed plans and fail to distract themselves from negative stimuli (Eysenck et al., 2007; Hildebrandt et al., 2016). These outcomes may further lead to difficulties with self-regulation (in particular, difficulties with emotional regulation) and social maladjustment, both of which may be key causes of Internet addiction. In addition, Davis (2001) proposed a cognitive-behavioral model of pathological Internet use where maladaptive cognition is hypothesized to be a vital cause of Internet addiction. The present study complements this model, indicating that self-regulation difficulties and social maladjustment caused by maladaptive cognition may be the major causes of Internet addiction.

Second, the results indicate that after controlling for self-regulation and self-inconsistency, cognitive flexibility can positively predict Internet addiction and plays a negative predictive role in the relationship between shyness and Internet addiction. This result indicates that without difficulties in self-regulation (especially emotional regulation) and social maladjustment, shy individuals may not develop severe Internet addiction. By contrast, research suggests that the use of network resources requires certain cognitive abilities (e.g., computer self-efficacy, cognitive spontaneity) because of the diversity and complexity of using such resources (Bozionelos, 1997; Durndell and Haag, 2002). For shy individuals, they may not be able to use computers flexibly and may even exhibit computer-related anxiety due to low cognitive flexibility caused by their impaired attention control system (Glass and Knight, 1988; Smith and Caputi, 2007). Therefore, the results revealed that after controlling for self-regulation and self-inconsistency, cognitive flexibility plays a negative predictive role in the relationship between shyness and Internet addiction.

Future research should further explore the relationship between cognitive flexibility and Internet addiction. The relationship between cognitive flexibility and Internet addiction may differ under various conditions.

### Role of Self-Regulation

Self-regulation (e.g., social-emotional regulation, cognition regulation) refers to individuals’ ability to effectively plan or modulate their behavior in consideration of an adaptive consequence (Montroy et al., 2016). The results of the present study revealed that owing to low cognitive flexibility, shy students may experience the predicament of self-regulation, which may be vital in the development of Internet addiction. Notably, shyness was not a direct predictor of self-regulation. The effect of shyness on self-regulation requires complete mediation from cognitive flexibility. Given the heavy effect of indirect path 2 (see Table 3., shyness→cognitive flexibility→ self-regulation→ Internet addiction, average indirect effect is 0.20), we may reasonably conclude that although cognitive flexibility is a crucial cause of Internet addiction in shy individuals, its effect on Internet addiction is exerted mainly through self-regulation. This results suggest that the dysfunctional link between cognitive factors and self-regulation may play an important role in the formation of Internet addiction in shy students.

### Role of Self-Inconsistency

Self-consistency and congruence are crucial concepts in Rogers’s personality theory (Rogers, 1959). Self-inconsistency reflects ability and emotion evaluation, self-consistency, and a sense of helplessness. In the present study, self-inconsistency was regarded as an indicator of social maladjustment. The results indicated that shyness, cognitive flexibility, and self-regulation may affect Internet addiction through self-inconsistency. This finding supported our hypothesis that because of self-defects and deficient cognitive and coping abilities (e.g., cognitive flexibility, self-regulation), shy individuals may face difficulties related to social maladjustment and easily develop Internet addiction. Specially, Shyness has been proven to be related to a series of social emotion and social adaptation problems. They may develop internalizing disorders, suffer peer rejection, experience loneliness, and experience difficulties adjusting to school environments (Chang et al., 2005; Yang et al., 2015). However, the anonymity and reduced cues that characterize the Internet might stimulate online self-disclosure. Hence, they may escape from social reality or fulfill social needs through excessive use of the Internet. These findings support the uses and gratifications theory and the social compensation hypothesis (McQuail et al., 1972; Sheldon, 2008). That is, shy students compensate themselves or release emotions through the Internet when suffering from social maladjustment.

The present study has several limitations. First, the cross-sectional design of this study provided limited information on the stability of the relation between shyness and Internet addiction. A longitudinal design should be implemented and may provide a better understanding of the relation between shyness and Internet addiction over time. Second, the self-report method of this study may lead to common method biases, thereby affecting the authenticity of the research. In addition, self-reporting method may be affected by perceptions of social desirability. Future studies should use multiple reporting methods to better explain the relation between shyness and Internet addiction. Furthermore, the correlational studies failed to reveal causal relationships among the variables. Future studies could use experimental methods to better explain the causal relationship between shyness and Internet addiction.

Despite its limitations, this study contributed substantially to the study and understanding of relation between personality and Internet addiction. Specifically, we established the relation between shyness and Internet addiction using a large sample from Chinese middle school students and determined the fully mediating effects of cognitive flexibility, self-regulation, and self-inconsistency on the relationship between shyness and Internet addiction. The results suggest that cognitive and coping abilities as well as social adjustment factors should be considered when designing interventions to help shy individuals overcome Internet addiction.

## Ethics Statement

Research activities were approved by Institutional Review Board of Shandong Normal University. Middle-school students (1320 non-clinical participants; mean age = 12.51 + 1.00; 50.2% female) were recruited from five schools in Shandong Province, East China, using a cluster sampling method. The questionnaire required approximately 30 min to complete. Written informed consents were obtained from the parents of all participants and received small gifts for their children’s participation.

## Author Contributions

YY collected the data and wrote the manuscript. HS wrote the manuscript. FG and YY revised the manuscript, and replied to comments.

## Conflict of Interest Statement

The authors declare that the research was conducted in the absence of any commercial or financial relationships that could be construed as a potential conflict of interest.

## References

[B1] Asendorpf J. B. (1990). The development of inhibition during childhood: evidence for a two- factor model. *Dev. Psychol.* 26 721–730. 10.1037//0012-1649.26.5.721

[B2] Bardi C. A. Brady M. F. (2010). Why shy people use instant messaging: loneliness and other motives. *Comp. Hum. Behav.* 26 1722–1726. 10.1016/j.chb.2010.06.021

[B3] Bozionelos N. (1997). Psychology of computer use: xlv. cognitive spontaneity as a correlate of computer anxiety and attitudes toward computer use. *Psychol. Rep.* 80 395–402. 10.2466/pr0.1997.80.2.395 9129359

[B4] Cañas J. Quesada J. F. Antolí A. Fajardo I. (2003). Cognitive flexibility and adaptability to environmental changes in dynamic complex problem-solving tasks. *Ergonomics* 46 482–501. 10.1080/0014013031000061640 12745698

[B5] Chak K. Leung L. (2004). Shyness and locus of control as predictors of internet addiction and internet use. *Cyberpsychol. Behav.* 7:559. 10.1089/1094931042403073 15667051

[B6] Chang L. Li K. K. Lei L. Liu H. Guo B. Wang Y. (2005). Peer acceptance and self-perceptions of verbal and behavioural aggression and withdrawal. *Int. J. Behav. Dev.* 29 49–57. 10.1080/01650250444000324

[B7] Cheek J. M. (1983). *The Revised Cheek and Buss Shyness Scale.* Wellesley. MA: WellesleyCollege.

[B8] China Internet Network Information Center [CNNIC] (2019). *The 43rd China Statistical Report on Internet Development.* Available at: http://www.cnnic.net.cn/hlwfzyj/hlwxzbg/hlwtjbg/201902/t20190228_70645.htm

[B9] Coplan R. J. Prakash K. O’Neil K. Armer M. (2004). Do you ”want” to play? distinguishing between conflicted shyness and social disinterest in early childhood. *Dev. Psychol.* 40 244–258. 10.1037/0012-1649.40.2.244 14979764

[B10] Coplan R. J. Rubin K. H. (2010). “Social withdrawal and shyness in childhood: History, theories, definitions, and assessments,” in *The Development of Shyness and Social Withdrawal*, eds Rubin K. H. Coplan R. J. (New York, NY: Guilford Press), 3–20.

[B11] Davis R. A. (2001). A cognitive-behavioral model of pathological internet use. *Comput. Hum. Behav.* 17 187–195. 10.1016/S0747-5632(00)00041-8

[B12] Durndell A. Haag Z. (2002). Computer self efficacy, computer anxiety, attitudes towards the internet and reported experience with the internet, by gender, in an east european sample. *Comput. Hum. Behav.* 18 521–535. 10.1016/s0747-5632(02)00006-7

[B13] Eysenck M. W. Derakshan N. Santos R. Calvo M. G. (2007). Anxiety and cognitive performance: attentional control theory. *Emotion* 7 336–353. 10.1037/1528-3542.7.2.336 17516812

[B14] Gao L. Zhang X. (2013). The relationship between family functioning and adolescents’ self-regulated learning: the mediation role of peer norms. *Stud. Psychol. Behav.* 11 153–157.

[B15] Glass C. R. Knight L. A. (1988). Cognitive factors in computer anxiety. *Cogn. Ther. Res.* 12 351–366. 10.1007/bf01173303

[B16] Greenfield P. M. Trumbull E. Keller H. Rothstein-Fisch C. Suzuki L. K. Quiroz B. (2006). “Cultural conceptions of learning and development,” in *Handbook of Educational Psychology*, 2nd Edn, eds Alexander P. A. Winne P. H. (Mahwah, NJ: Lawrence Erlbaum), 675–692.

[B17] Hammick J. K. Lee M. J. (2014). Do shy people feel less communication apprehension online? the effects of virtual reality on the relationship between personality characteristics and communication outcomes. *Comput. Hum. Behav.* 33 302–310. 10.1016/j.chb.2013.01.046

[B18] Hayes A. (2013). Introduction to mediation, moderation, and conditional process analysis. *J. Educ. Measure.* 51 335–337. 10.1111/jedm.12050 28385036

[B19] Hildebrandt L. K. Mccall C. Engen H. G. Singer T. (2016). Cognitive flexibility, heart rate variability, and resilience predict fine-grained regulation of arousal during prolonged threat. *Psychophysiology* 53 880–890. 10.1111/psyp.12632 26899260

[B20] Hormes J. M. Kearns B. Timko C. A. (2014). Craving facebook? behavioral addiction to online social networking and its association with emotion regulation deficits. *Addiction* 109:2079. 10.1111/add.12713 25170590

[B21] Huan V. S. Ang R. P. Chong W. H. Chye S. (2014). The impact of shyness on problematic internet use: the role of loneliness. *J. Psychol.* 148 699–715. 10.1080/00223980.2013.825229 25175891

[B22] Hutteman R. Denissen J. Asendorpf J. Aken M. (2009). Changing dynamics in problematic personality: a multiwave longitudinal study of the relationship between shyness and aggressiveness from childhood to early adulthood. *Dev. Psychopathol.* 21 1083–1094. 10.1017/S0954579409990058 19825258

[B23] Kaess M. Durkee T. Brunner R. Carli V. Parzer P. Wasserman C. (2014). Pathological internet use among European adolescents: psychopathology and self-destructive behaviours. *Eur. Child Adolesc. Psychiatry* 23 1093–1102. 10.1007/s00787-014-0562-7 24888750 PMC4229646

[B24] Lavin M. J. Yuen C. N. Weinman M. Kozak K. (2004). Internet dependence in the collegiate population: the role of shyness. *Cyberpsychol. Behav.* 7 379–383. 10.1089/cpb.2004.7.379 15331024

[B25] Li C. Dang J. He S. Li H. (2013). Shyness and loneliness: the multiple mediating effects of self-efficacy. *Acta Psychol. Sin.* 45 1251–1260. 10.1007/BF00584098

[B26] Liu L. (2008). Study on relationship between internet addiction disorder and type c behavior pattern of college students. *China J. Health Psychol.* 16 370–372.

[B27] Lu X. Yeo K. J. (2015). Pathological Internet use among malaysia university students: risk factors and the role of cognitive distortion. *Comput. Hum. Behav.* 45 235–242. 10.1016/j.chb.2014.12.021

[B28] Maroldo G. K. (1986). Shyness and alcohol response expectancy hypothesis: social situations. *Am. Psychol.* 41 1386–1387. 10.1037/0003-066X.41.12.1386

[B29] Martin M. M. Anderson C. M. Thweatt K. S. (1998). Aggressive communication traits and their relationships with the cognitive flexibility scale and the communication flexibility scale. *J. Soc. Behav. Personal.* 13 531–540.

[B30] Martin M. M. Rubin R. B. (1995). A new measure of cognitive flexibility. *Psychol. Rep.* 76 623–626. 10.2466/pr0.1995.76.2.623

[B31] McKenna K. Bargh J. (2000). Plan 9 from cyberspace: the implications of the internet for personality and social psychology. *Personal. Soc. Psychol. Rev.* 4 57–75. 10.1207/s15327957pspr0401_6

[B32] McQuail D. Blumler J. G. Brown J. R. (1972). “The television audience: Revised perspective,” in *Sociology of Mass Communications*, ed. McQuail D. (Harmondsworth: Penguin Books), 135–165.

[B33] Montroy J. J. Bowles R. P. Skibbe L. E. Mcclelland M. M. Morrison F. J. (2016). The development of self-regulation across early childhood. *Dev. Psychol.* 52:1744. 10.1037/dev0000159 PMC5123795 27709999

[B34] Morahan-Martin J. Schumacher P. (2000). Incidence and correlates of pathological Internet use among college students. *Comput. Hum. Behav.* 16 13–29. 10.1016/S0747-5632(99)00049-7

[B35] Nelson L. J. Coyne S. M. Howard E. Clifford B. N. (2016). Withdrawing to a virtual world: associations between subtypes of withdrawal, media use, and maladjustment in emerging adults. *Dev. Psychol.* 52:933. 10.1037/dev0000128 27148777

[B36] Orr E. S. Sisic M. Ross C. Simmering M. G. Arseneault J. M. Orr R. R. (2009). The influence of shyness on the use of facebook in an undergraduate sample. *Cyberpsychol. Behav.* 12 337–340. 10.1089/cpb.2008.0214 19250019

[B37] Preacher K. J. Hayes A. F. (2004). Spss and sas procedures for estimating indirect effects in simple mediation models. *Beh. Res. Methods Instr. Comput.* 36 717–731. 10.3758/bf03206553 15641418

[B38] Qi B. Zhao B. Wang K. Liu H. (2013). Revision and preliminary application of cognitive flexibility scale for college students. *Stud. Psychol. Behav.* 4 57–75. 10.1207/S15327957PSPR0401_6

[B39] Rogers C. R. (1959). “A theory of therapy, Personality, and interpersonal relationship as developed in the client-centered framework,” in *Psychology: A Study of Science*, ed. Koch S. (New York, NY: McGraw-Hill), 184–256.

[B40] Rogers C. R. (1961). “A tentative scale for the measurement of process in psychotherapies,” in *Contemporary Psychotherapies*, ed. Stein M. P. (New York, NY: Free Press), 184–256.

[B41] Rubin K. H. Coplan R. J. Bowker J. C. (2009). Social withdrawal in childhood. *Ann. Rev. Psychol.* 60:141 10.1146/annurev.psych.60.110707.163642 PMC3800115 18851686

[B42] Saunders P. L. Chester A. (2008). Shyness and the internet: social problem or panacea? *Comput. Hum. Behav.* 24 2649–2658. 10.1016/j.chb.2008.03.005

[B43] Scealy M. Phillips J. G. Stevenson R. (2002). Shyness and anxiety as predictors of patterns of internet usage. *Cyberpsychol. Behav.* 5 507–515. 10.1089/109493102321018141 12556113

[B44] Scharlott B. W. Christ W. G. (1995). Overcoming relationship-initiation barriers: the impact of a computer-dating system on sex role, shyness, and appearance inhibitions. *Comput. Hum. Behav.* 11 191–204. 10.1016/0747-5632(94)00028-g

[B45] Scherer K. (1997). College life online: healthy and unhealthy internet use. *J. Coll. Stud. Dev.* 38 655–665. 10.1023/A:1003013826225

[B46] Selfhout M. H. Branje S. J. Delsing M. Bogt T. F. T. Meeus W. H. (2009). Different types of internet use, depression, and social anxiety: the role of perceived friendship quality. *J. Adolesc.* 32 819–833. 10.1016/j.adolescence.2008.10.011 19027940

[B47] Sheldon P. (2008). The relationship between unwillingness-to-communicate and students’ facebook use. *J. Media Psychol. Theor. Methods Appl.* 20 67–75. 10.1027/1864-1105.20.2.67

[B48] Smith B. Caputi P. (2007). Cognitive interference model of computer anxiety: implications for computer-based assessment. *Comput. Hum. Behav.* 23 1481–1498. 10.1016/j.chb.2005.07.001

[B49] Walker A. M. Rablen R. A. Rogers C. R. (1960). Development of a scale to measure process changes in psychotherapy. *J. Clin. Psychol.* 16 79–85. 10.1002/1097-4679(196001)16:1<79::aid-jclp2270160129>3.0.co;2-k

[B50] Xu Y. Farver J. A. Yu L. Zhang Z. (2009). Three types of shyness in chinese children and the relation to effortful control. *J. Personal. Soc. Psychol.* 97:1061. 10.1037/a0016576 19968419

[B51] Yang F. Chen X. Wang L. (2015). Shyness-sensitivity and social, school, and psychological adjustment in urban chinese children: a four-wave longitudinal study. *Child Dev.* 86:1848. 10.1111/cdev.12414 26331958

[B52] Young C. M. Y. Lo B. C. Y. (2012). Cognitive appraisal mediating relationship between social anxiety and internet communication in adolescents. *Personal. Individ. Diff.* 52 78–83. 10.1016/j.paid.2011.09.001

[B53] Young K. (1996). Internet addiction: the emergence of a new clinical disorder. *Cyber Psychol. Behav.* 3 237–244. 10.1089/cpb.1998.1.237 22271405

[B54] Young K. (1999). Cyber-disorders: the mental health concern for the new millennium. *CyberPsychol. Behav.* 2 475–479. 10.1089/cpb.1999.2.475 19178220

[B55] Young K. S. de Abreu N. C. (2011). *Internet Addiction: A Handbook and Guide to Evaluation and Treatment*, eds Hoboken Edn. NJ: John Wiley &Sons Inc.

[B56] Yu Y. Sun H. Gao F. Han Lei H. (2019). Neurocognitive deficits in shy college students: an event-related potential analysis of the p3 component evoked by evaluations of others. *Personal. Individ. Diff.* 138 40–47. 10.1016/j.paid.2018.09.014

[B57] Zhang H. Li D. Li X. (2015). Temperament and problematic Internet use in adolescents: a moderated mediation model of maladaptive cognition and parenting styles. *J. Child Fam. Stud.* 24 1886–1897. 10.1007/s10826-014-9990-8

[B58] Zhao J. Kong F. Wang Y. (2012). Self-esteem and humor style as mediators of the effects of shyness on loneliness among chinese college students. *Personal. Individ. Diff.* 52 686–690. 10.1016/j.paid.2011.12.024

[B59] Zhou P. (2015). The self-congruence of minority secondary school students and its effect on their learning engagement. *Chin. J. Special Educ.* 9 90–96. 10.3969/j.issn.1007-3728.2015.09.014

